# Major Traditional Probiotics: Comparative Genomic Analyses and Roles in Gut Microbiome of Eight Cohorts

**DOI:** 10.3389/fmicb.2019.00712

**Published:** 2019-04-09

**Authors:** Guangwen Luo, Bailiang Li, Cailu Yang, Yutang Wang, Xin Bian, Wan Li, Fei Liu, Guicheng Huo

**Affiliations:** ^1^Key Laboratory of Dairy Science, Ministry of Education, College of Food Science, Northeast Agricultural University, Harbin, China; ^2^Department of Ultrasound, Maternal and Child Health Hospital of Dapeng New District, Shenzhen, China

**Keywords:** major traditional probiotics species, comparative genomic analyses, complementarity potentials, roles in gut microbiome, cohorts

## Abstract

Modulating gut microbiota to promote host health is well recognized. Therefore, people consume dietary products containing traditional probiotics in wishing to improve their health, and they need more research-based advices on how to select products with suitable probiotic species. Probiotic designers are sometimes confused about how to design precision products for different consumers by taking advantages of different probiotic species’ strengths. Additionally, large-scale analyses on traditional probiotic complementarity potentials and their roles in gut microbiome related to common diseases are not well understood. Here, we comprehensively analyzed 444 genomes of major traditional probiotic (sub) species (MTPS, *n* = 15) by combining one newly sequenced genome with all of the public NCBI-available MTPS-related genomes. The public human fecal metagenomic data (*n* = 1,815) of eight cohorts were used to evaluate the roles of MTPS, compared to other main gut bacteria, in disease association by examining the species enrichment direction in disease group or the control group. Our work provided a comprehensive genetic landscape and complementarity relations for MTPS and shed light on personalized probiotic supplements for consumers with different health status and the necessity that researchers and manufactures could explore novel probiotics as well as traditional ones.

## Introduction

Traditional probiotics bacteria are mainly lactic acid bacteria (LAB) (e.g., *Lactobacillus*, *Lactococcus*, and *Bifidobacteria*). They are a group of Gram-positive bacteria that produce lactic acid as a main end product and have been generally considered as safe for human consumption. It also has been shown that probiotics administrated closer to the first dose of antibiotics can reduce the risk of *Clostridium difficile* infection in hospitalized adults by more than 50% (Shen et al., 2017). Another traditional probiotic is *Bacillus* bacteria, mostly *Bacillus subtilis*, which was reported to have abolished colonization of *Staphylococcus aureus*, a dangerous pathogen in a rural Thai population by inhibiting *S. aureus* quorum sensing (Piewngam et al., 2018). Specific strains associated with health benefits had been used as probiotics, establishing a huge consumption market. However, reports of clinical infection or adverse effects, and the spread of antibiotic resistance genes, have raised concerns about safety. A case of an elderly diabetic patient with a complicated liver abscess and bacteremia from the consumption of *Lactobacillus paracasei* probiotic has been reported and confirmed by strain identification (Pararajasingam and Uwagwu, 2017). Probiotic therapy should be carefully selected in hemodialysis patients since probiotic supplements failed to reduce uremic toxins and inflammations (Borges et al., 2018). Compared to spontaneous post-antibiotic recovery, probiotics induced a significant delay and persistently incomplete indigenous stool/mucosal microbial reconstitution and host transcriptome recovery toward homeostatic configuration (Suez et al., 2018).

Gratefully, public research data and approaches provide a good foundation for further inspection of the host-microbiome relationships. The total amount of sequenced bacterial genomes has continued to expand markedly over the past few years (Land et al., 2015). Intestinal microbes have been explored for several decades, and the investigations of the role of human gut microbiota have drawn much attention about diseases, such as obesity, diabetes, liver diseases, and even cancer and neurodegenerative diseases. There is a trend that the human gut microorganisms are considered a resource of novel therapies (Cani, 2018). What is more, metagenome-wide association studies (MWAS) is a research method to survey the correlations between the human microbiota and many non-communicable diseases (Wang and Jia, 2016). Such many public bacterial genomes data, case-control cohort metagenomes data and advanced research methods make it possible to systematically compare the functional potential and safety factors of MTPS, as well as the detection of their abundance and evaluation of their impacts on the gut microbiomes.

In this article, 444 strains (of species labeled in the Chinese domestic market probiotics products and one children OTC drug) and 1,815 public human gut metagenomic data from eight cohorts were recruited, in the aims to describe the species diversity, potential risk gene components, strain complementarity and to assess the impacts of MTPS on gut microbiomes, shedding light on personalized probiotics supplement for consumers and urgent need that researchers and manufactures could explore more novel probiotics as well as traditional ones.

## Materials and Methods

### Genomes Recruitment

We collected most of the species names from labels of commercially available yogurt products and one children OTC drug (Omitting product names to avoid commercial benefits conflict). All associated genomes were downloaded from NCBI (updated to March 19th, 2018) by searching species keywords in the URL^1^. Items with “single cell,” “Partial,” “derived from environmental source,” “metagenome,” “too small,” “low contig N,” “frameshifted,” “missing tRNA genes,” or “abnormal gene to sequence ratio” were eliminated in this work in order to avoid low-quality genomes.

The *Lactobacillus acidophilus* (*Lb. acidophilus*) KLDS1.0901 was isolated in our laboratory. A small fragment library of 400 bp insert size and a mate-pair large fragment library of 6 Kbp insert size were constructed, respectively, and sequenced on the Illumina Genome Analyzer II, HiSeq/MiSeq platform. Approximately 150× clean reads were produced by filtering low quality (>40% ambiguous “N” bases or >40% bases of quality <20 in one or both of the paired-end reads), low complexity and duplicated reads, then were assembled into scaffolds by SOAPdenovo2 (version r240) (Luo et al., 2012). This Whole Genome Shotgun project of *Lb. acidophilus* KLDS1.0901 has been archived at DDBJ/ENA/GenBank under the accession MNPO00000000. The version involved in this study is version MNPO01000000.

Genome quality was further assessed. Firstly, more rigorous five high-quality draft genome criteria from the Human Microbiome Project (HMP) (Human Microbiome Jumpstart Reference Strains et al., 2010) was adopted to ensure the assembly quality. The criteria were (i) 98% of the total assembly must be present in contigs (>500 bp), (ii) the scaffold N50 must be more than 20 kb, (iii) the contig N50 must be more than 20 kb, (iv) the average contig length must be more than 10 kb, and (v) more than 90% of the “core genes” (Callister et al., 2008) must be present in the assembly. Secondly, the “complete” and “contamination” of genomes were checked using CheckM (v1.0.12) (Parks et al., 2015). Only genomes with “complete” >95% and “contamination” <5% were recruited. Thirdly, average nucleotide identity (ANI) (Richter and Rossello-Mora, 2009) was also employed to remove the far-distance genomes which may be mis-distributed to the species. The pairwise ANI should be >93% for each pair of genomes within the same species. For ANI-based whole genome comparison among genomes, identity alignments were done with BLAST (version 2.2.26) (Mount, 2007). ANI was counted for every genome pair using the method as previously described (Zhang et al., 2014) basing on the BLAST results. Another same alignment was done by exchanging the query genome and the reference genome. The pairwise ANI value was the average value of the two alignments. The average amino acid identity (AAI) calculation was same as ANI, except replacing nucleotide sequence with amino acid sequence.

### Pan-Genome Analyses

A pan-genome for a (sub) species cluster consists of core genes shared by all the recruited genomes, plus dispensable genes consisting of accessory gene partially shared in members and strain-specific genes unique to single members (Tettelin et al., 2005). To identify orthologous genes for the strains in each species, CD-HIT (v4.6.6-2016-0711) (Li and Godzik, 2006) was applied to cluster genes as described previously (Kim et al., 2017), except that the thresholds of 95% protein sequence similarity and 90% alignment length for shorter sequence.

### Phylogenetic Analyses

A group of 40 universally conserved single-copy gene coding proteins in prokaryotic microbes was applied for the construction of a phylogenetic tree. The marker genes were deduced by specI (v1.0) (Mende et al., 2013) and aligned using prank (v.170427) (Loytynoja, 2014). Alignments were truncated by trimal (v1.4.1) (Capella-Gutierrez et al., 2009) and concatenated into one amino acid sequence by in-house scripts. Maximum-likelihood phylogenetic trees for all the genomes were constructed in RAxML (version 8.2.11) (Stamatakis, 2014), and were visualized on iTOL (Letunic and Bork, 2016) online.

### Functional Assignments and KEGG Module Complementarity *in silico* Calculation

Genes were identified using Genemark (version 2.6r) (Besemer and Borodovsky, 2005). The translated amino acid sequences of coding genes were aligned with RAPSearch (v2.23) (Ye et al., 2011) (default options, except for -s f -e 1e-2 -v 100 -u 2) against Kyoto Encyclopedia of Genes and Genomes (KEGG version 76) (Kanehisa and Goto, 2000) (query match length >=50%) or with BLASTp (default options, except for -e 1e-2 -F T -b 100 -K 1 -a 1 -m 8) against databases of Antibiotic Resistance Genes Database (ARDB) (Liu and Pop, 2009) (both query and subject match length >=40%, with identity more than the ARDB-recommended thresholds), Virulence Factor Database (VFDB) (Chen et al., 2016) (query match length >=50%, with identity >=60%), Carbohydrate-Active enZYmes Database (CAZy) (Lombard et al., 2014) (query match length >=50%, with identity >=60%).

The KEGG module integrity was defined as matched key/essential KO number divided by all key/essential KO number required by this module. To learn the KEGG module complementarity of every genome pair, all the KOs of every genome pair were merged to calculate the improvement of module integrity.

### Gut Microbiome Profiling and Co-abundance Network Analyses

The clean reads (filtering out low-quality reads and host-derived DNA reads) from each cohort were, respectively, profiled (taxonomy at species level) by MetaPhlAn2 (v2.2.0) (Segata et al., 2012) (default options, except setting – ignore_viruses – ignore_eukaryotes –ignore_archaea). The clean reads from ACVD cohorts were also, mapped [SOAP2 (v2.22) (Li et al., 2009), with default parameters] to the gene set of each species or all genes of the target strain. The mapping ratio for each gut microbiome sample was defined as mapped reads divided by total reads of each sample.

Pearson correlation (*p*-value <0.05, correlation coefficient cutoff, >0.4 if positive, <-0.1 if negative) for species relative abundances (derived from MetaPhlAn2) in all samples (without consideration of health status) among the enriched species for each cohort were obtained, and co-abundance networks (all enriched species were showed only) were visualized with Cytoscape (v3.6.1) (Shannon et al., 2003) software.

## Results

### Diverse Openness Level of Pan-Genomes Could Be Related to Multiple Forces

The completeness, purity and quality of genomes can influence the pan-genome results (Kim et al., 2017), thus the quality control was necessary for all the recruited genomes. A total of 502 genomes belonging to 15 (sub)species were downloaded from NCBI as of March 19th, 2018, and 444 high-quality genomes (including our newly sequenced strain *Lb. acidophilus* KLDS1.0901) were retained after the removal of low quality, impure, and mis-allocated genomes recognized by HMP five genome draft standards, CheckM and intra-species ANI. In particular, 38 strains of *Bacillus subtilis* subsp. *subtilis* (*Ba. subtilis*), 19 strains of *Bifidobacterium longum* subsp. *infantis* (*Bi. infantis*), 35 strains of *Bifidobacterium longum* subsp. *longum* (*Bi. longum*), 22 strains of *Bifidobacterium animalis* subsp. *lactis* (*Bi. lactis*), 20 strains of *Lb. acidophilus* (containing KLDS1.0901), 15 strains of *Lactobacillus delbrueckii* subsp. *bulgaricus* (*Lb. bulgaricus*), 33 strains of *Lactobacillus casei* (*Lb. casei*), 31 strains of *Lactobacillus fermentum* (*Lb. fermentum*), 25 strains of *Lactobacillus gasseri* (*Lb. gasseri*), 16 strains of *Lactobacillus helveticus* (*Lb. helveticus*), 52 strains of *Lactobacillus paracasei* (*Lb. paracasei*), 9 strains of *Lactobacillus plantarum* subsp. *plantarum* (*Lb. plantarum*), 25 strains of *Lactococcus lactis* subsp. *cremoris* (*Lc. cremoris*), 61 strains of *Lactococcus lactis* subsp. *lactis* (*Lc. lactis*), and 43 strains of *Streptococcus thermophilus* (*Sc. thermophilus*) were retained to illuminate the pan-genomes, core genes and strain-specific genes to understand the characteristic of the pan-genomes of each species. Detailed genomic information for all the strains are summarized in Supplementary Table S1.

**FIGURE 1:**
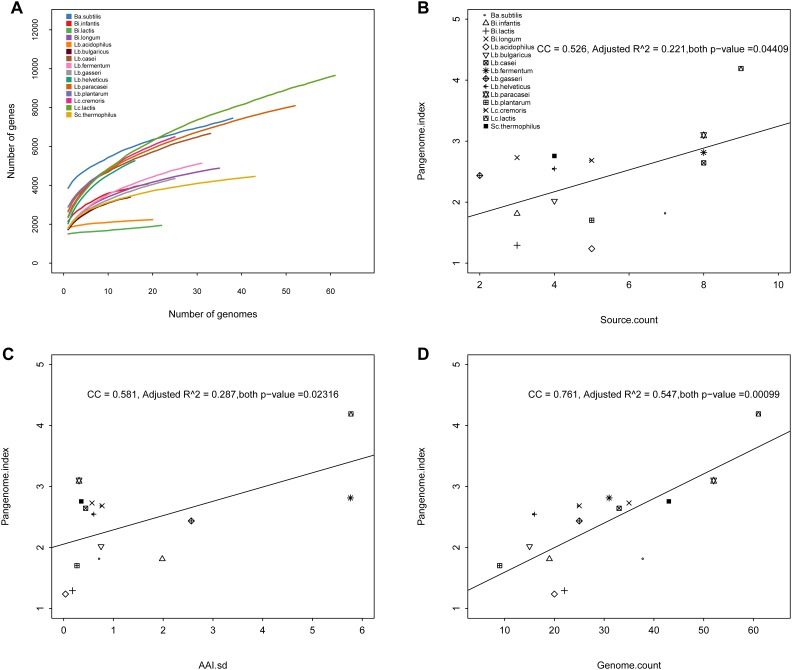
Pan-genome rarefaction and pan-genome index. **(A)** The pan-genome rarefaction curve of 15 species. **(B)** Correlation between pan-genome index and source count of each species, CC: correlation coefficient. **(C)** Correlation between pan-genome index and intra-species amino acid identity (AAI) SD. **(D)** Correlation between pan-genome index and genome count of each species.

In order to quantitate the openness (genetic diversity) of pan-genome, pan-genome index (PI) was defined in this study at the first time, namely, average gene number divided by gene number of pan-genome. The openness of pan-genomes could be divided into three levels (Figure 1A) for these involved species. Level A, relatively conserved, PI<=1.5, including *Lb. acidophilus* (PI 1.24) and *Bi. lactis* (PI 1.29). Level B, half-open, 1.5<PI<=3, including *Lb. plantarum* (PI 1.7), *Bi. infantis* (PI 1.81), *Ba. subtilis* (PI 1.82), *Lb. bulgaricus* (PI 2.02), *Lb. gasseri* (PI 2.44), *Lb. helveticus* (PI 2.55), *Lb. casei* (PI 2.64), *Lc. cremoris* (PI 2.68), *Bi. longum* (PI 2.73), *Sc. thermophilus* (PI 2.76), *Lb. fermentum* (PI 2.81). Level C, relatively open, including *Lb. paracasei* (PI 3.1), and *Lc. lactis* (PI 4.19).

The species isolated from different hosts are enriched in specific gene sets, indicating host-specific adaptation (Sun et al., 2015b). That is to say, genetic diversity is related to the ecological niche, so we further surveyed relationship including this among MTPS. The original isolation sources were collected as much as possible for these strains. The association analysis results showed that PI was weakly positively associated [correlation coefficient (CC) 0.526, R square 0.221] with intra-species isolation source count (Figure 1B). PI was also weakly positively associated (CC 0.581, R square 0.287) with intra-species genome number (Figure 1D). PI was weakly positively associated (CC 0.761, R square 0.547) with intra-species AAI distance standard deviation (AAI SD, Figure 1C). In summary, the correlation was growing and being strengthened along with isolation source, genome number and AAI distance SD. Interestingly, the R square was improved to 0.585 (*p*-value 0.005) when combining all the three factors, implying that many factors would influence the PI and should be taken into consideration together. The reason for this might be that the bacteria could display genomic diversity in assorted niche sites for survival by insert, deletion, single nucleotide polymorphisms, structure variation, and lateral gene transfer, resulting in ever-increasing genetic diversity (Duranti et al., 2016).

### Phylogenetic Tree Cluster on Species Level Even on Subspecies Level

**FIGURE 2:**
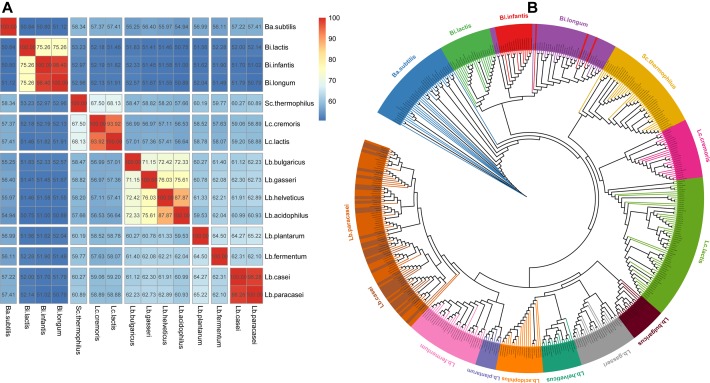
Amino acid identity matrix and phylogenetic tree. **(A)** AAI matrix at species level. The number in the cell indicated the AAI value of the two species. Clusters are separated by blank lines **(B)** Phylogenetic tree for 444 strains.

To investigate the distance of 15 species, pairwise AAI of the core gene of each species were computed. Strains from the same microbial species share >95% AAI and ANI (Thompson et al., 2013). Around 95% ANI is the recommended threshold for species discrimination (Mende et al., 2013). Both *Bi. infantis* and *Bi. longum* belong to *Bi. longum*, and their AAI (98.4%) is greater than 95%, corroborating they were the same species at molecular level. *Lb. paracasei* and *Lb. casei* belong to different species, but their AAI (98.28%) is greater than 95%, showing that they should be the same species at molecular level. In some taxonomy annotation software, such as MetaPhlAn2, *Lb. paracasei* and *Lb. casei* are treated as same species with the name *Lactobacillus_casei_paracasei*. *Lc. cremoris* and *Lc. lactis* belong to *Lc. lactis*, but their AAI (93.92%) is less than 95%, showing that they should be different species at molecular level. At the 70% AAI cutoff, 15 species could be separated into 8 clusters (Figure 2A). The ANI matrix for 444 strains were summarized in Supplementary Table S2.

Further, the phylogenetic relationships among these strains of 15 species were also inspected based on 40 universally conserved single-copy proteins (Mende et al., 2013). We observed that the 15 species are also divided into 8 clusters (Figure 2B), which was in agreement with the clustering with AAI. As shown on the tree, *Lb. paracasei* and *Lb. casei* blended with each other, demonstrating they should be the same species again. *Bi. infantis* and *Bi. longum* (both isolated from human gut) also blended with each other, which implied that they are not fully differentiated due to their similar lifestyle adaptation (Yu et al., 2018).

### Potential Risk Factors Differ Among Species and Are Elevated in Groups of Unfriendly Isolation Sources

To investigate the risk factors (Ronholm et al., 2016) of all 444 strains, VFDB and ARDB genes were considered and the annotation results were compared for every species pair to find species-enriched/specific features. In total, these strains contained 11 VFDB genes and 18 ARDB genes (Supplementary Table S3), suggesting they had moderate risk factors. Concretely speaking, *Ba. subtilis* harbored the most specific VFDB genes (VFG001373, VFG011430, VFG045350) and ARDB genes (ardb_1014, ardb_1206, ardb_1207, ardb_129, ardb_2757, ardb_295, ardb_3001, ardb_3001, ardb_365, ardb_676), while *Bi. lactis* harbored none. VFG000077 (ATP-dependent Clp protease proteolytic subunit), VFG000080 (ATP-dependent protease), VFG002182 (UDP-galactopyranose mutase) are prevalent for most of the species. VFG000079 (endopeptidase Clp ATP-binding chain C) was shared by *Ba. subtilis*, *Lb. casei*, and *Lb. paracasei*. VFG002165 (endocarditis specific antigen) and ardb_1371 (bacitracin) are shared by *Lb. casei* and *Lb. paracasei*. VFG013286 (UDP-glucose 4-epimerase) was shared by *Bi. infantis* and *Bi. longum*. ardb_2198 (bacitracin) was shared by *Lb. bulgaricus*, *Lb. fermentum*, and *Lb. plantarum*. ardb_2980 (bacitracin) was shared by *Lc. lactis* and *Sc. thermophilus*. VFG002187 (glycosyl transferase, group 2 family protein), ardb_1049 (fluoroquinolone) and ardb_751 (unknown function) are specific for *Lc. cremoris*. ardb_1050 (fluoroquinolone), ardb_2979 (bacitracin), and ardb_750 (unknown function) are specific for *Lc. lactis*. VFG005767 (3-ketoacyl-ACP-reductase CylG) was specific for *Sc. thermophilus*.

Additionally, risk factors between relatively friendly group (with isolation source of such as milk, cheese, yogurt, beer, human gut, food) and relatively unfriendly group (with isolation source of such as patient’s specimen, soil, blood, saliva, silage) were tested by unpaired Wilcoxon-rank sum test. As a result, the strains isolated from unfriendly environments harbored much more risk factors (Supplementary Figure S1, ^∗^*p* < 0.05, ^∗∗^*p* < 0.01), indicating that it is recommended to choose strains with a clear safe source when using them in food industry (Ronholm et al., 2016), such as yogurt starter.

### Relatively Enhanced Functions Differ Among Species, Even Subspecies

**FIGURE 3:**
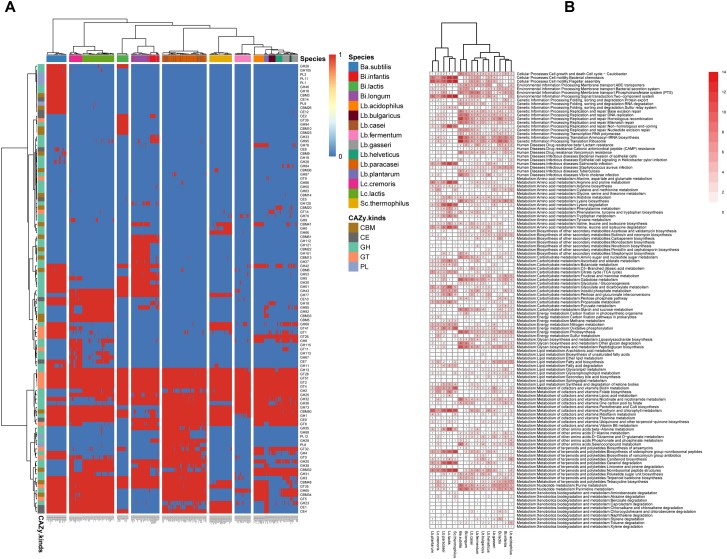
Different relatively enhanced function features among species. **(A)** Heat map illustrating the distribution of carbohydrate-active enzyme genes. **(B)** Different relatively enhanced function of KEGG pathway map. The number in the cell indicated how many other species are significantly weaker than this species in this function (*q*-value <0.05, FDR-controlled Wilcoxon rank-sum test).

The CAZy database is a extensively used resource associated with the enzymes that construct and breakdown complex carbohydrates and glycoconjugates (Cantarel et al., 2009). From the viewpoint of CAZy annotation results (Figure 3A), *Ba. subtilis*, *Bi. infantis*, *Bi. longum*, *Lb. plantarum*, *Lc. cremoris*, and *Lc. lactis* harbored leading capability of carbohydrate metabolism, while *Lb. fermentum*, *Lb. helveticus*, and *Lb. bulgaricus* harbored lagging capability relatively. There were also species-specific CAZy module, such as GT39, GH94, CBM10 and CBM23 for *Bi. lactis*, CBM61 and GH112 for *Bi. infantis* and *Bi. longum* (both are subspecies of *Bi. longum*), GH66 for *Sc. thermophilus*. To find all the enhanced CAZy modules for 15 species, all the modules were tested by unpaired Wilcoxon-rank sum test for every species pair, the detailed results were provided in Supplementary Table S3, which is helpful to compare carbohydrate metabolism characteristic of 15 species.

KEGG is another well-known database resource for understanding advanced functions and tools of the biological system from molecular-level information (Kanehisa and Goto, 2000). From the insight of KEGG annotation results (Figure 3B, KEGG pathway map; Supplementary Figure S2, KEGG pathway module), we could find the differences of 15 species in metabolism capability other than carbohydrate metabolism, such as Cellular processes, Environmental information processing, Genetic information processing, Amino acid metabolism, Lipid metabolism, Vitamins metabolism and energy metabolism. There were two main clusters, cluster 1 contained *Lb. plantarum*, *Lc. cremoris*, *Lb. paracasei*, *Lc. lactis*, and *Sc. thermophilus*, cluster 2 contained *Ba. subtilis*, *Bi. longum*, *Lb. casei*, *Lb. fermentum*, *Lb. bulgaricus*, *Lb. helveticus*, *Lb. gasseri*, *Bi. lactis*, *Bi. infantis*, and *Lb. acidophilus*. Cluster 1 was dominated by *Sc. thermophilus*, *Lc. lactis*, and *Lb. paracasei*, and was superior in functions associated with Bacterial chemotaxis, Flagellar assembly, Two-component system, Arginine and proline metabolism, Lysine degradation, Tryptophan metabolism, Valine, leucine, and isoleucine degradation, Oxidative phosphorylation, Biotin metabolism, Porphyrin and chlorophyll metabolism, Beta-Alanine metabolism, Iosynthesis of siderophore group non-ribosomal peptides, Geraniol degradation, Non-ribosomal peptide structures. Cluster 2 was dominated by *Ba. subtilis* and *Bi. longum*, and was superior in functions associated with Cell cycle-caulobacter, Phosphotransferase system, Homologous recombination, Aminoacyl-tRNA biosynthesis, Ribosome, Vancomycin resistance, Lysine biosynthesis, Peptidoglycan biosynthesis, Other glycan degradation, Nicotinate and nicotinamide metabolism, Purine metabolism and pyrimidine metabolism.

At the subspecies level, there were still a few differences (between *Lc. cremoris* and *Lc. lactis*, or between *Bi. infantis* and *Bi. longum*). *Lc. cremoris* was superior in CAZy functions associated with GH5, GT41, GT1, GT8, and in KEGG functions associated with Cysteine and methionine metabolism, Sulfur metabolism, Fatty acid biosynthesis, Nicotinate and nicotinamide metabolism, Tetracycline biosynthesis and Purine metabolism, while *Lc. lactis* was superior in CAZy functions associated with GH85, GH2, and in KEGG functions associated with Non-homologous end-joining, Arginine and proline metabolism, Lysine degradation, Valine, leucine and isoleucine degradation, Ascorbate and aldarate metabolism, Glyoxylate and dicarboxylate metabolism. *Bi. infantis* was superior in CAZy functions associated with GH33, GH95, GH29, and in KEGG functions associated with Sulfur relay system, *S. aureus* infection, Cysteine and methionine metabolism, Butirosin and neomycin biosynthesis, Fructose and mannose metabolism, Inositol phosphate metabolism, Fatty acid biosynthesis, Glycerolipid metabolism, Tetracycline biosynthesis, while *Bi. longum* was superior in CAZy functions associated with CBM41, GH121, CBM22, GH101, CBM13, GH27, CBM6, GH30, CBM32, GH31, and in KEGG functions associated with DNA replication, Homologous recombination, Mismatch repair, Nucleotide excision repair, *Vibrio cholerae* infection, Amino sugar and nucleotide sugar metabolism, Starch and sucrose metabolism, Peptidoglycan biosynthesis, Other glycan degradation.

The function components difference served as a foundation for species complementation and implied that species (even subspecies) diversity and characters should be taken into account for co-cultivation (Stewart, 2012).

### Farther Distance for Genomes, Higher Complementarity for Function

**FIGURE 4:**
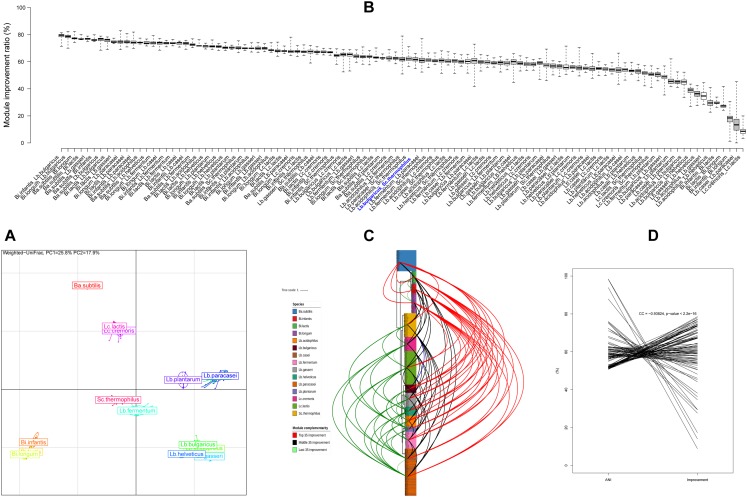
KEGG module feature and complementarity. **(A)** Principal components analysis based on the KEGG module integrity profile for all strains. Isolates on the first and second principal components (PC1 and PC2) are plotted by nodes. Lines link strains of the same species, and colored circles pack the strains near the barycenter for each species. **(B)** Module complementarity at strain level. **(C)** Module complementarity at species level showed on the phylogenetic tree with three groups; red lines, top 1/3 combination; black lines, medium combination; green lines, last 1/3 combinations; blue lines, the classical combination of *Lb. bulgaricus* and *Sc. thermophilus*. **(D)** Correlation between ANI and module complementarity at species level.

The complementarity of KEGG module was *in silico* computed for each strain. As a result, 444 surveyed strains form eight distinct clusters (Figure 4A), which was in line with the previous cluster results derived from CAZy and phylogenetic tree, revealing that there were significant differences not only in genomic features but also in functions between these strains at cluster level (mostly at species level).

In addition, the complementarity improvement ratios of KEGG module were counted for each pair of strains belonging to different species. Consequently, 90,543 combinations (Supplementary Table S4) were obtained, and were displayed by the average improvement ratio at species level sequentially from high to low (Figure 4B, 105 boxes) and also displayed on the phylogenetic tree at species level (Figure 4C, 105 connection lines) and also plotted against ANI values of every species pair (Figure 4D, 105 connection lines). The complementarity improvement ratio varied over a broad range (from 0 to 82.95%), illustrating that strain heterogeneities were prevalent. The farther distance between branches, the higher complementarity for species. The smaller ANI values, the higher complementarity for species. *Lb. bulgaricus* and *Sc. thermophilus* is a classical traditional combination for yogurt starter, whereas their complementarity only laid the middle position (No. 50) of all these complementarities.

Altogether, there were huge potentials for developing more excellent multi-strain probiotic products, and it seems like that the strain with farther genomic distance will improve the function complementarity at higher levels.

### Notably Different Roles in the Human Cohort Gut Microbiomes

To investigate the relative abundances and roles of MTPS in human gut microbiomes, public fecal metagenomics sequencing clean reads (*n* = 1,815) from obesity (Liu et al., 2017) (*n* = 200, 105 control and 95 case, without weight-loss intervention), type 2 diabetes (Qin et al., 2012) (T2D, *n* = 368, 185 control and 183 case), atherosclerotic cardio-vascular disease (Jie et al., 2017) (ACVD, *n* = 385, 171 control and 214 case), Crohn’s disease (He et al., 2017) (CD, *n* = 102, 53 control and 49 case), colorectal adenoma-carcinoma (Feng et al., 2015) (CRC, *n* = 147, 57 control and 90 case), liver cirrhosis (Qin et al., 2014) (LC, *n* = 237, 114 control and 123 case), ankylosing spondylitis (Wen et al., 2017) (AS, *n* = 207, 114 control and 93 case), and rheumatoid arthritis (Zhang et al., 2015) (RA, *n* = 169, 74 control and 95 case) human cohorts were downloaded to form species profiles by MetaPhlAn2. Detailed species enrichment direction, species abundance rank and network-linkages rank in each cohort gut microbiomes are in Supplementary Table S5.

**FIGURE 5:**
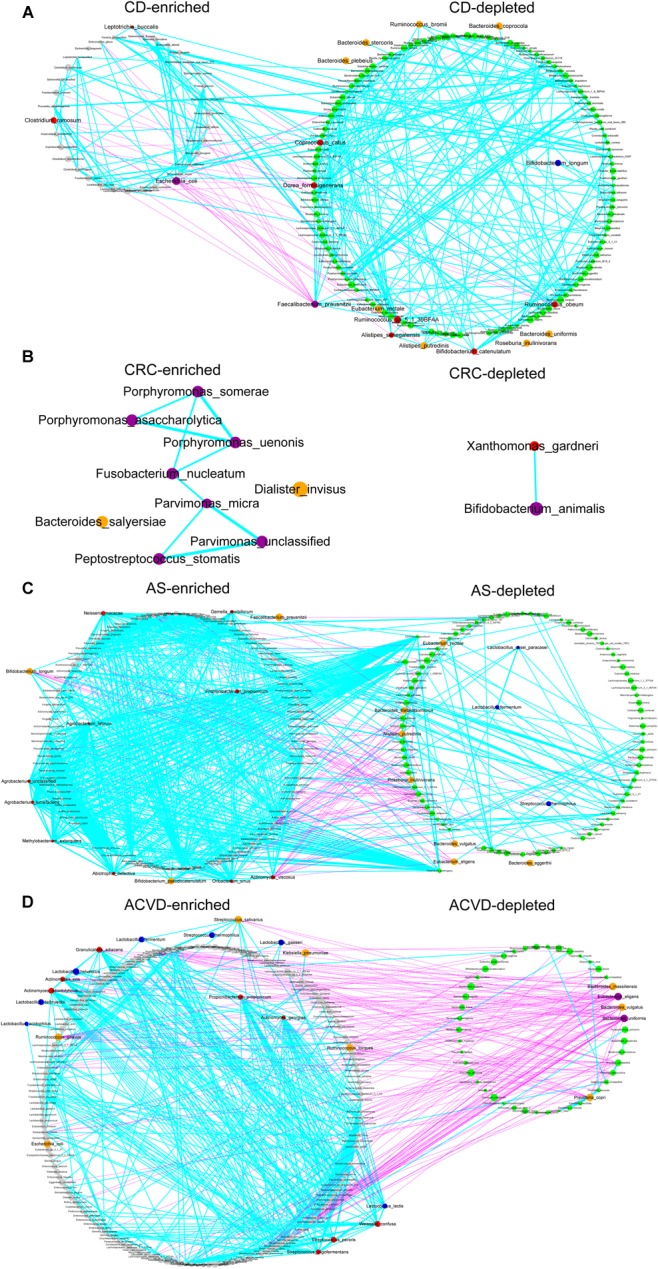
Traditional probiotics roles in the cohort gut microbiome. **(A)** The cohort of CD. **(B)** The cohort of CRC. **(C)** The cohort of AS. **(D)** The cohort of ACVD. Co-abundance network of species significantly enriched in case or control subjects. Left, network in individuals with disease (case-enriched); right, network in controls (case-depleted). Species with significantly different relative abundances between the groups are displayed (*q*-value <0.05, FDR-controlled Wilcoxon rank-sum test). Gray circles, case-enriched; green circles, control-enriched; purple circles, species with top 10 edges and top 10 abundance; red circles, species with top 10 edges; orange circles, species with top 10 abundance; blue circles, MTPS. The size of each circle indicates the mean relative abundance of species (1/10, 1/100, …, 1/10^∗∗^6, <1/10^∗∗^6). Please see Supplementary Table S5 for more detailed information on enriched or depleted species. Cyan edges means positive correlations; pink edges means negative correlations. The edge width declines along the absolute value of the Pearson cc: thick edges, |cc| >=0.7; medium, 0.4 <=|cc| <0.7; thin, 0.1 < |cc| < 0.4.

CD is an inflammatory bowel disease, and specific pathogenic bacteria that potentially cause CD have been anchored, such as adherent-invasive *Escherichia coli* (He et al., 2017). In our data, there were 35 CD-enriched species and 130 CD-depleted species (Figure 5A), indicating CD might be mainly induced by the deficiency of beneficial strains. *E. coli*, *Clostridium ramosum*, and *Leptotrichia buccalis* might be the main contributors in CD-enriched group, while *Faecalibacterium prausnitzii*, *Ruminococcus* sp. 5_1_39BFAA, *Dorea formicigenerans*, *Coprococcus catus*, *Ruminococcus obeum* might be the main contributors in CD-depleted groups. It is interesting to find that *B. longum* was also enriched in the CD-depleted group with 13th network-rank and 21st abundance (Supplementary Table S5).

CRC is one of the top three most frequently diagnosed cancers worldwide and a chief cause of cancer mortality. The gut microbiota is believed to be directly involved in colorectal carcinogenesis (Feng et al., 2015). There were 9 CRC-enriched species and 2 CRC-depleted species (Figure 5B). *Porphyromonas uenonis*, *Porphyromonas somerae*, *Fusobacterium nucleatum*, *Parvimonas micra*, *Porphyromonas asaccharolytica*, *Peptostreptococcus stomatis* might be the main contributors in CRC-enriched group, while *B. animalis* was one of the two CRC-depleted group members. Altogether, CRC might be mainly induced by the excessive growth of harmful strains.

AS is a chronic, systemic, inflammatory autoimmune disease featured by the inflammation of the axial skeleton, the peripheral joints, and the attachments of ligaments and enthuses (Wen et al., 2017). There were 108 AS-enriched species and 96 AS-depleted species (Figure 5C), indicating AS might be a complicated disease with both excessive growth of harmful strains and deficiency of beneficial strains. *Lb. fermentum*, *Lb. casei paracasei*, and *St. thermophilus* of MTPS were the AS-depleted group members, while *B. longum*, which showed benefits in CD disease, was enriched in AS-enriched group with potentially adverse effects.

ACVD is a cardiometabolic disease associated with high morbidity and mortality (Jie et al., 2017). There were 169 ACVD-enriched species and 46 ACVD-depleted species (Figure 5D), indicating ACVD might be a complicated disease with both mainly excessive growth of harmful strains and remarkable deficiency of beneficial strains. Unexpectedly, *Lb. fermentum*, *Lb. acidophilus*, *Lb. delbrueckii*, *Lb. gasseri*, *Lb. helveticus*, *Lc. lactis*, and *St. thermophilus* of MTPS were the ACVD-enriched group members. On the other hand, *Bacteroides uniformis*, *Eubacterium eligens*, *Prevotella copri*, *Bacteroides vulgatus*, and *Bacteroides massiliensis* were in ACVD-depleted group with top network-linkages and top abundances, suggesting that they might be the main contributors and the novel potential candidate probiotics for ACVD prevention and remission.

LC is an advanced-stage liver disease originating from acute or chronic liver injury, comprising alcohol abuse, obesity, and hepatitis virus infection (Qin et al., 2014). There were 246 LC-enriched species and 113 LC-depleted species (Supplementary Figure S3a), indicating LC might be a complicated disease with both mainly excessive growth of harmful strains and remarkable deficiency of beneficial strains. *Veillonella* unclassified, *Veillonella parvula*, *Haemophilus parainfluenzae*, *Haemophilus influenzae* and so on were the main contributors in the LC-enriched group. Unexpectedly, *Lb. fermentum*, *Lb. delbrueckii*, *Lb. gasseri*, and *St. thermophilus* (all four were ACVD-enriched) of MTPS were the LC-enriched group members, showing a very similar phenomenon between LC and ACVD. On the other hand, *Bilophila* unclassified, *Alistipes senegalensis*, *Alistipes putredinis*, *Coprococcus catus* were in LC-depleted group with top network-linkages, suggesting that they might be the main contributors and the potential candidate novel probiotics for LC prevention and remission.

T2D is a complicated disorder affected by both genetic and environmental components, and has become a major public health problem worldwide (Qin et al., 2012). There were 23 T2D-enriched species and 21 T2D-depleted species (Supplementary Figure S3b), indicating that T2D might not be as complicated as AS, ACVD, and LC. *Clostridiales bacterium 1_7_47FAA*, *Eggerthella lenta*, *Lactobacillus mucosae*, *C. ramosum*, *Clostridium citroniae* and so on might be the main contributors in the T2D-enriched group. Unexpectedly, *Lb. helveticus* of MTPS was the non-primary member in the T2D-enriched group. On the other hand, *Faecalibacterium prausnitzii*, *Actinobacillus* unclassified, *Roseburia inulinivorans*, and *Eubacterium rectale* were in the T2D-depleted group with top network-linkages, suggesting that they might be the main contributor and the potential candidate for novel probiotics for T2D prevention and remission. Although *H. parainfluenzae*, *Haemophilus sputorum*, and *Haemophilus paraphrohaemolyticus* were in T2D-depleted group with top network-linkages, even some in top abundance, their roles should be seriously recognized since *H. parainfluenzae* was enriched in LC.

Obesity has become a global epidemic and is a major risk factor for type 2 diabetes, cardiovascular diseases and certain cancers. Evidence has accumulated that the gut microbiota is an important environmental factor contributing to obesity by altering host energy harvest and storage (Cox et al., 2015). There were 60 Obesity-enriched species and 50 Obesity-depleted species (Supplementary Figure S3c), indicating Obesity might be not so complicated as to AS, ACVD and LC. *Streptococcus mitis*, *Streptococcus mitis/oralis/pneumoniae*, *Streptococcus pneumoniae*, *Ruminococcus gnavus*, *Abiotrophia defectiva*, *Granulicatella elegans*, *Streptococcus oligofermentans*, *Gemella sanguinis*, and *Streptococcus cristatus* might be the main contributors in Obesity-enriched group. On the other hand, *Alistipes shahii*, *A. putredinis*, and *Odoribacter splanchnicus* were in Obesity-depleted group with top network-linkages, suggesting that they might be the main contributor and the potential candidate novel probiotics for Obesity prevention and remission. It is interesting to see that MTPS showed no significant effects in both groups.

RA is a common autoimmune disorder that causes progressive disability and systemic complications. There were 4 RA-enriched species and 11 RA-depleted species (Supplementary Figure S3d), indicating RA might be not so complicated as to AS, ACVD and LC. *Atopobium parvulum*, *Actinomyces oris*, *Actinomyces viscosus*, and *Lactobacillus salivarius* might be the main contributors in RA-enriched group. On the other hand, *Megamonas hypermegale*, *Megamonas* unclassified, *Megamonas funiformis*, *Megamonas rupellensis*, *Xanthomonas gardneri*, *Pseudomonas protegens*, and *Pseudomonas extremaustralis* were in RA-depleted group with top network-linkages, suggesting that they might be the main contributor and the potential candidate novel probiotics for RA prevention and remission. It is interesting to see that MTPS showed no significant effects in both groups, as same as Obesity.

After manual inspection, we found that the enriched species enrichment directions in eight cohorts in this study were basically in agreement with the reports of literatures from which the public data derived. Except for *B. animalis* in CRC, MTPS were not the main contributors in disease association, even make no influences (in Obesity and RA cohorts), with respect to relative abundance, co-abundance networks and enriched MTPS proportion in the total enriched species in each cohort.

To get a stronger proof for the reliabilities of species enrichment directions and the relative abundance of MTPS based on the species annotation by MetaPhlAn2, the host-free clean reads of ACVD cohorts as a representative were directly mapped onto each gene set of the 15 species, respectively. Difference of reads mapping ratio between control and case were tested by unpaired Wilcoxon-rank sum test. As shown in Supplementary Figure S4a, at species level, *Bi. infantis* and *Bi. longum* were high-abundance, while other species were very low-abundance in human gut microbiomes. The enrichment direction for above-mentioned ACVD-enriched species among MTPS, such as *Lb. fermentum*, *Lb. acidophilus*, *Lb. bulgaricus*, *Lb. gasseri*, *Lb. helveticus*, *Lc. Lactis*, and *St. thermophilus*, were in line with that by MetaPhlAn2.

To further investigate whether each strain of high-abundance species show significances of reads mapping ratio between control and case, all strains belonging to *Bi. infantis* and *Bi. longum* were, respectively, mapped by ACVD reads. As a result, there were no significant differences (adjusted *p-value* > 0.05, unpaired Wilcoxon-rank sum test) between control group and case group at strain level (Supplementary Figure 4b).

## Discussion

Designing multi-strain probiotics products combining pre-existing strains with ever-increasing newly isolated ones is challenging, since it is costly to conduct physiological and biochemical experiments by combining them together and personalized probiotics (Zmora et al., 2018) consumption would make a future trend. As many as possible, this study collected the public genome sequences of the bacterial names deduced from the labels of probiotics products and one children OTC drug on the Chinese supermarket and conducted comparative genomic analyses. We recruited genomes with high-quality standard (Chain et al., 2009), which are suited to mine all associated phylogenetic and functional information, and enable analyses for genome regions of interest or whole genomes (Sun et al., 2015a). Firstly, we defined PI and investigate the impact factors on the openness of pan-genome, showing that there were notably differences in the openness level of pan-genome in different species, which might be affected by multi factors encompassing the strain isolation source, the genome characteristics and the number of genomes within species. Thus, when selecting strains, the strains with open pan-genome or subspecies should be looked into more carefully than other relatively conservative species. Secondly, we compared the safety scores with respect to virulence factor, antibiotic resistance genes between groups with friendly isolation sources (e.g., milk, dairy or specimen from healthy people) or unfriendly sources, showing that the strains isolated from the friendly ecological niche harbored significant lower virulence factors and antibiotic resistance genes, while it must be admitted that the reported isolation source of any involved species might not necessarily be where it evolved (Sun et al., 2015a). Thirdly, we highlighted the species/strain enriched/specific advantage of metabolic capacity and quantified the combination complementarity based on KEGG module function, demonstrating that farther genetic distance resulted in a greater possibility to achieve stronger combinations. However, co-cultivation experiments are still needed in the future (Marmann et al., 2014).

With respect to the species (MetaPhlAn2) enrichment analyses and co-abundance association analyses, some of MTPS showed benefits to digestive tract-associated diseases, such as CD (*Bi. longum*) and CRC (*B. animalis*), and solid organ-associated diseases, such as LC (*Ba. subtilis*) and AS (*Lb. casei_paracasei*, *Lb. fermentum*, *Sc. thermophilus*). Whereas some of MTPS showed potentially adverse effects to cardiometabolic diseases, such T2D (*Lb. helveticus*) and ACVD (*Lb. acidophilus*, *Lactobacillus_delbrueckii*, *Lb. fermentum*, *Lb. gasseri*, *Lb. helveticus*, *Lactococcus_lactis*, *Sc. thermophilus*), and solid organ associated diseases, such as LC (*Lactobacillus_delbrueckii*, *Lb. fermentum*, *Lb. gasseri*, *Lb. helveticus*, *Sc. thermophilus*) and AS (*Bifidobacterium_longum*). Additionally, with respect to the reads mapping results, most of MTPS were relatively more abundant in ACVD group than control group, which was basically in line with the result by MetaPhlAn2. However, there were too few mapped reads for most of the MTPS, therefore it remains to be seen whether this trend will apply to other experiments (and whether these species will indeed be prevalent in the human gut). And even if this phenomenon will be corroborated, the impacts on human health might be very limited due to their rather low proportion and their limited role, compared to other key players among gut microbiota. It is worth noting that both the differences in the gut microbiota abundance but also the differences in its metabolism and functions need to be taken into consideration in evaluating their contributions to the physiopathology of related diseases (Villanueva-Millan et al., 2015). In any case, it seems that the patients of T2D, LC, AS or ACVD might pay more attention to the selection of live probiotic products with best-suited strains.

On the other hand, the metagenomics-based microbiome abundance comparison between case and control subjects might lead to the false claim that a bacterium is causally correlated with the protection or the promotion of a disease (Cani, 2018). In fact, environmental factors such as dietary habits, drug treatments, intestinal motility, and stool frequency and consistency are all factors that influence the composition of the microbiota and should be considered (Cani, 2018). Insights into the impacts of spectrum and power of MTPS on the host still need to be strengthened in the future.

All in all, although the causal role of MTPS in gut microbiome of eight cohorts, especially T2D, ACVD, LC, and AS, still needs further investigation, our analyses exhibit a global correlation between gut microbiome (including MTPS) and the protection or the promotion of common diseases. This study provides conceptual advances that lays the foundation for future applications in personalized probiotics, which must consider both the species/strain heterogeneity and individual heterogeneity.

## Author Contributions

GH and GL designed the study. GL, BL, CY, YW, XB, WL, and FL performed the experiments and analyzed the data. GL wrote the manuscript.

## Conflict of Interest Statement

The authors declare that the research was conducted in the absence of any commercial or financial relationships that could be construed as a potential conflict of interest.

## Footnotes

1 ftp://ftp.ncbi.nlm.nih.gov/genomes/genbank/assembly_summary_genbank.txt

## Supplementary Material

FIGURE S1 Comparison of risk factors between relatively friendly group and relatively unfriendly group. Unpaired wilcoxon-rank sum test, ^∗^p < 0.05, ^∗∗^p < 0.01.

FIGURE S2 Different relatively enhanced function of KEGG pathway module. The number in the cell indicated how many other species are significantly weaker than this species in this function (q-value < 0.05, FDR-controlled Wilcoxon rank-sum test).

FIGURE S3 Traditional probiotics roles in another four cohort gut microbiome. **(a)** The cohort of LC. **(b)** The cohort of T2D. **(c)** The cohort of Obesity. **(d)** The cohort of RA. The notes for the networks are same as that in Figure 5.

FIGURE S4 Difference of reads mapping ratio between control and case in ACVD at species or strain level. **(a)** Species level for 15 species. **(b)** Strain level within Bi.infantis and Bi.longum. Unpaired wilcoxon-rank sum test, ^∗^p < 0.05, ^∗∗^p < 0.01, ^∗∗∗^p < 0.001, ^∗∗∗∗^p < 0.0001.

TABLE S1 Detailed genomic information for all the 444 strains.

TABLE S2 The ANI matrix for all the 444 strains.

TABLE S3 Caption for Sheet “risk.factors”: Species-enriched VFDB and ARDB gene counts and annotations. Caption for Sheet “CAZy”: Species-enriched CAZy gene counts and annotations.

TABLE S4 The complementarity improvement ratios of KEGG module for each pair strains belonging to different species.

TABLE S5 Detailed species enrichment direction, species abundance rank and network-linkages rank in each cohort gut microbiomes.
