# Supplementary figures and images for: Major Traditional Probiotics: Comparative Genomic Analyses and Roles in Gut Microbiome of Eight Cohorts
Source: Front Microbiol. 2019 Apr 9;10:712. doi: 10.3389/fmicb.2019.00712 (PMC6465617; doi:10.3389/fmicb.2019.00712)

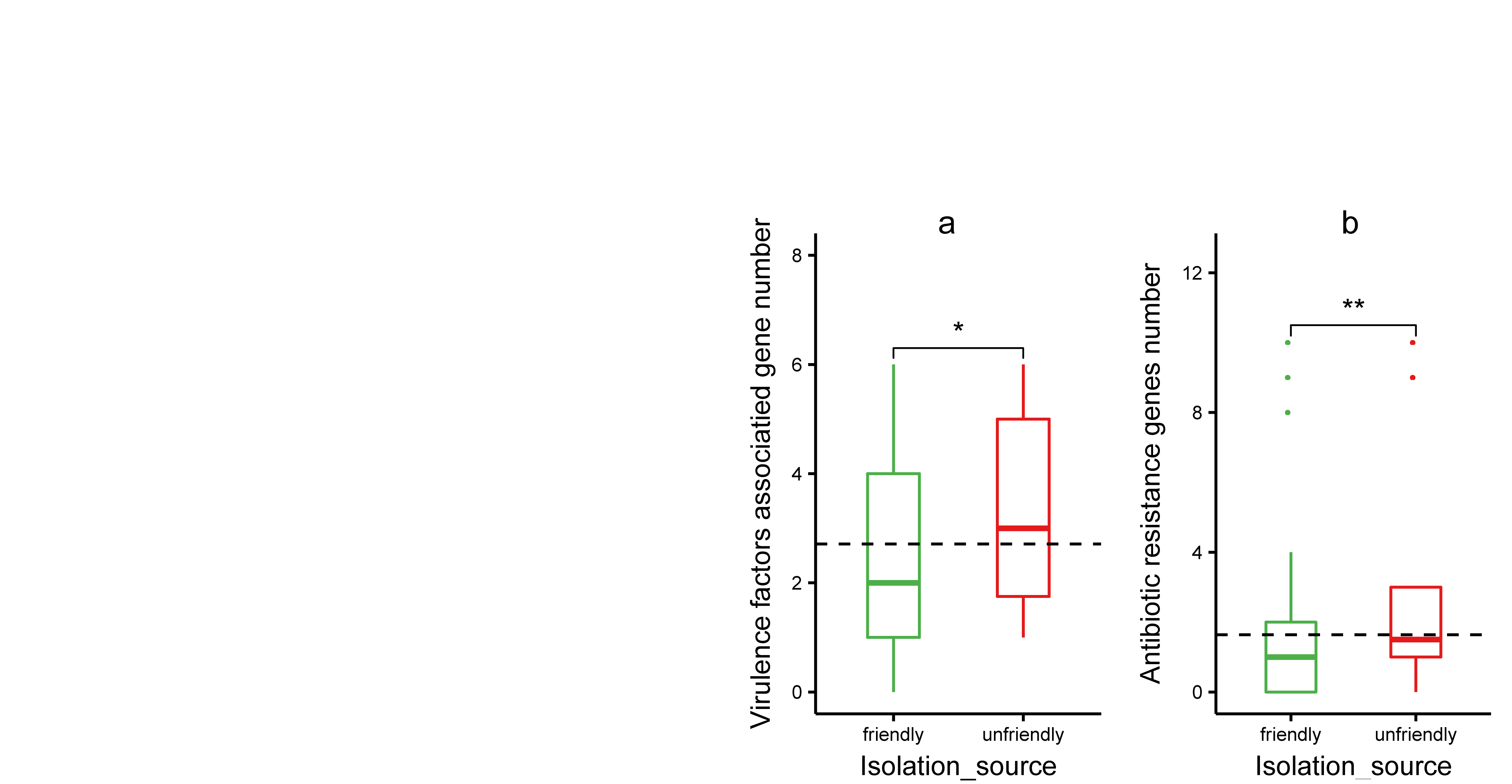

Supplement: FIGURE S1 — Comparison of risk factors between relatively friendly group and relatively unfriendly group. Unpaired wilcoxon-rank sum test, ∗p < 0.05, ∗∗p < 0.01. [file Image_1.TIF]

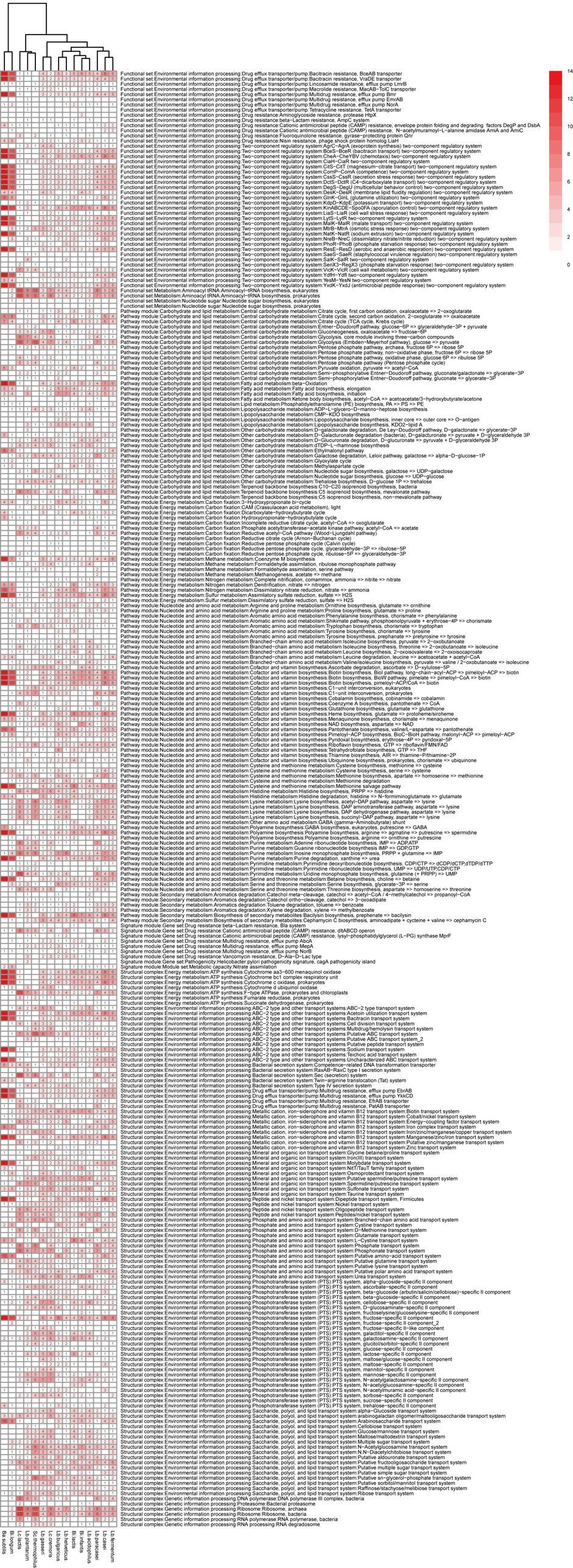

Supplement: FIGURE S2 — Different relatively enhanced function of KEGG pathway module. The number in the cell indicated how many other species are significantly weaker than this species in this function (q-value < 0.05, FDR-controlled Wilcoxon rank-sum test). [file Image_2.TIF]

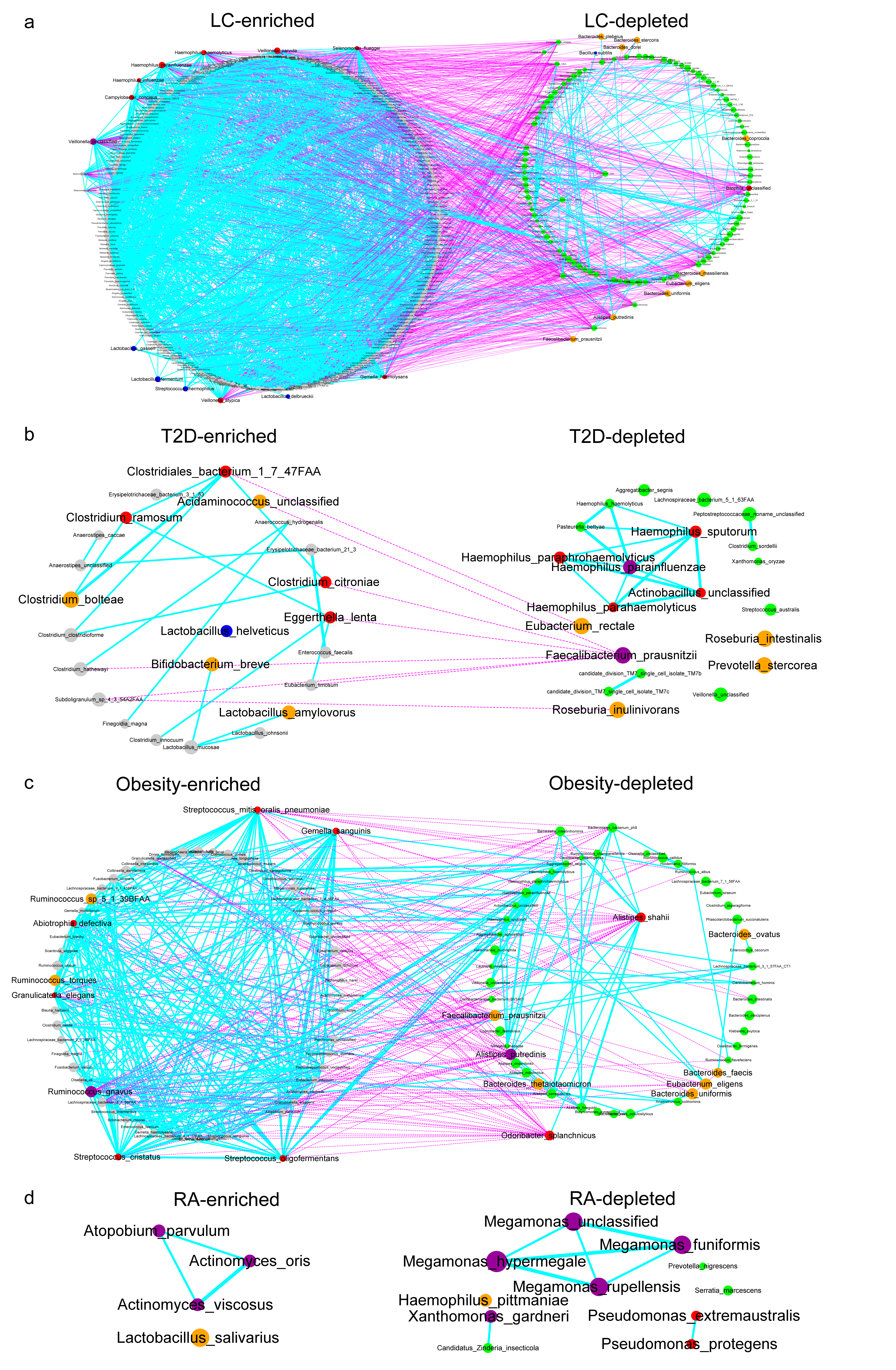

Supplement: FIGURE S3 — Traditional probiotics roles in another four cohort gut microbiome. (a) The cohort of LC. (b) The cohort of T2D. (c) The cohort of Obesity. (d) The cohort of RA. The notes for the networks are same as that in Figure 5. [file Image_3.TIF]

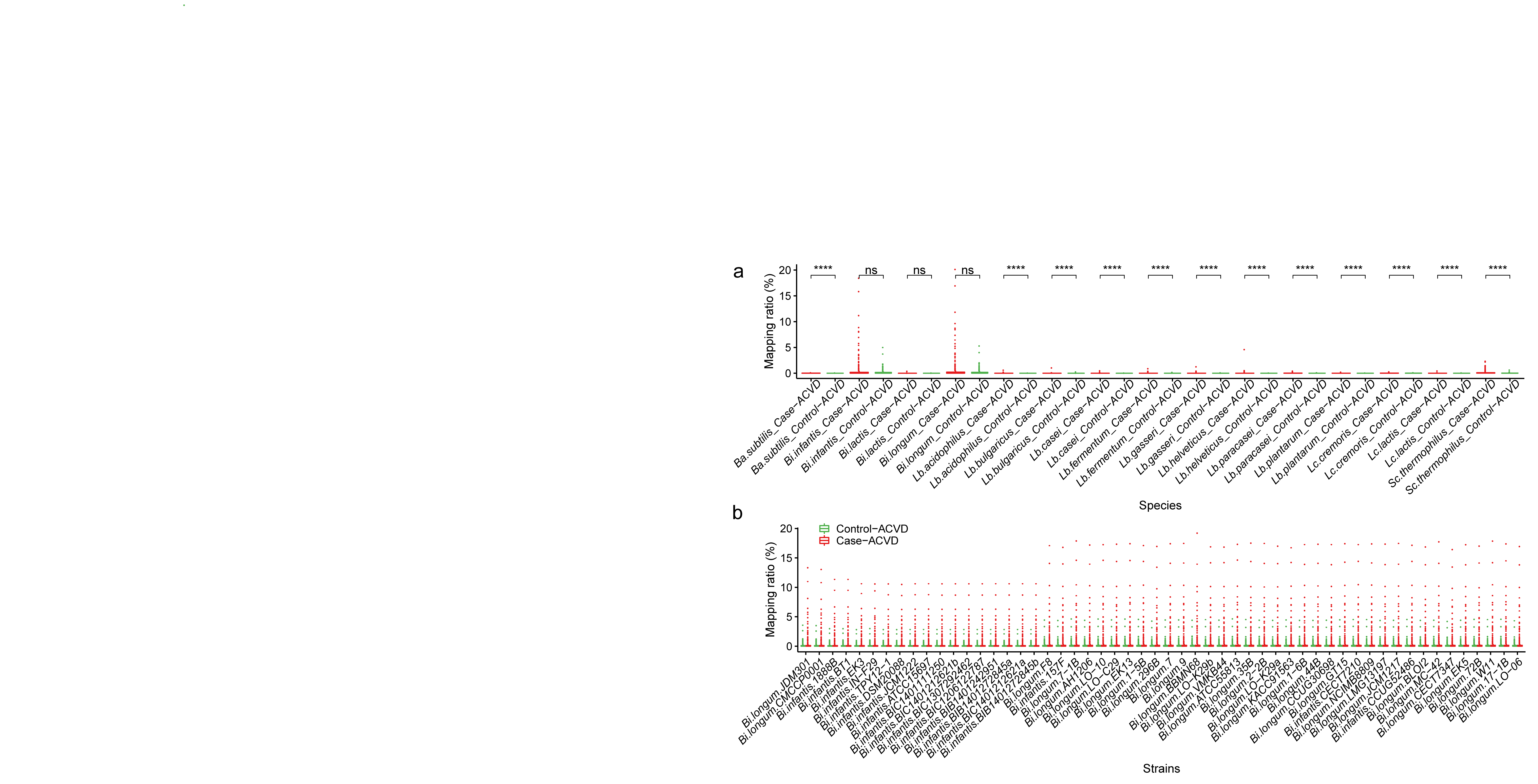

Supplement: FIGURE S4 — Difference of reads mapping ratio between control and case in ACVD at species or strain level. (a) Species level for 15 species. (b) Strain level within Bi.infantis and Bi.longum. Unpaired wilcoxon-rank sum test, ∗p < 0.05, ∗∗p < 0.01, ∗∗∗p < 0.001, ∗∗∗∗p < 0.0001. [file Image_4.TIF]
